# Splenic infarction after endoscopic glue injection for gastric varices: proportion, exploratory correlates, and clinical outcomes from a propensity score-matched analysis

**DOI:** 10.3389/fmed.2026.1823208

**Published:** 2026-09-07

**Authors:** Bin Liang, Jun Lin, Zhijun Yang, Jiaxing Liu, Hongmei Wang, Jinlong Li, Dongmei Wang

**Affiliations:** 1 Department of Gastroenterology, The Fifth People's Hospital of Ganzhou, Ganzhou, China; 2 Ganzhou Institute of Liver Disease, Ganzhou, China; 3 Department of Pathology, Ganzhou Cancer Hospital, Ganzhou, China

**Keywords:** cyanoacrylate glue, endoscopic complications, gastric varices, propensity score matching, splenic infarction

## Abstract

**Background and aim:**

Splenic infarction after endoscopic cyanoacrylate injection for gastric varices (GV) is poorly characterized. We estimated its proportion among computed tomography (CT)-evaluable patients and explored clinical features and short-term outcomes.

**Methods:**

We retrospectively screened 255 adult patients undergoing endoscopic glue injection for GV between January 2018 and February 2025. Eligible patients had CT within 30 days and no pre-existing infarction or alternative causes; imaging was clinically selected rather than protocol-mandated. Expanded 1:4 propensity score matching included clinical, laboratory, and index-session endoscopic features. Exploratory Firth regression addressed sparse events and complete separation.

**Results:**

Of 183 CT-evaluable patients, 16 (8.7%) had radiologically confirmed splenic infarction. After matching, 16 cases and 64 controls were analyzed, although residual imbalance remained. All cases had ≥2 previous injection sessions versus 15.6% of matched controls, producing complete separation; the Firth estimate was therefore not considered a stable effect size. Exploratory models showed statistical signals for repeated injections, larger splenic diameter, large spontaneous portosystemic shunts, and greater index-session glue volume. These features may represent advanced portal hypertension and treatment complexity rather than independent risk factors. Most cases (93.8%) were managed conservatively. Thirty-day mortality and rebleeding did not differ, whereas hospital stay was longer (median, 14 vs. 10 days; *p* = 0.008).

**Conclusions:**

Splenic infarction was identified in 8.7% of this selected CT-evaluable cohort. The observed features should be regarded as exploratory clinical markers, not causal or actionable predictors. Imaging selection, residual imbalance, 16 events, complete separation, and wide uncertainty preclude incidence estimation, precise effect-size interpretation, or individual risk prediction.

## 1 Introduction

Gastric varices (GV), particularly those located in the fundus (IGV1 and GOV2), pose a significant clinical challenge in the management of portal hypertension (1). Compared to esophageal varices, they are less common but often larger, carry a higher risk of severe bleeding, and are associated with greater mortality (2, 3). The endoscopic management of GV differs substantially from that of esophageal varices. Endoscopic variceal ligation (EVL), the mainstay for esophageal varices, is often less effective and technically difficult for large, tortuous GV due to the risk of causing massive, uncontrollable hemorrhage (4). Similarly, conventional sclerotherapy carries a high risk of ulceration and rebleeding (5).

It is within this context that endoscopic injection of cyanoacrylate glue (such as N-butyl-2-cyanoacrylate) has emerged as the first-line endoscopic treatment. Upon injection into the varix, the liquid glue rapidly polymerizes upon contact with blood, forming a solid cast that effectively obliterates the lumen of the vessels (6). This mechanism provides immediate mechanical occlusion, leading to highly effective hemostasis in active bleeding and durable eradication for prophylaxis (7, 8). Consequently, endoscopic glue injection is established as the preferred endoscopic modality for bleeding GV and for primary or secondary prophylaxis in high-risk patients (9, 10).

Despite its high success rate, the procedure is not without risks. While common adverse events like post-procedural fever, abdominal pain, and ulceration are generally self-limiting, serious complications, particularly systemic embolism, remain a significant concern (11, 12). Embolic events can occur when glue migrates into the systemic circulation, most frequently through spontaneous portosystemic shunts (SPSS), which are present in a substantial proportion of patients with advanced cirrhosis and portal hypertension. Embolism to critical sites such as the pulmonary, cerebral, or coronary arteries, though rare, can be catastrophic (13, 14).

Splenic infarction represents a specific and less commonly reported form of embolic complication following endoscopic glue injection (15). The presumed mechanism involves the inadvertent entry of glue into the splenic circulation, either via retrograde flow through the splenic vein or, more plausibly, through large SPSS (e.g., splenorenal or gastrorenal shunts) that provide a direct pathway from the GV to the splenic vasculature (16, 17). However, due to its perceived rarity, the true incidence, precise risk factors, and clinical impact of this complication are not well characterized. Existing literature is limited to isolated case reports and small series, lacking robust data from larger cohorts (18). Consequently, there is a lack of awareness among clinicians, and no evidence-based strategies exist for risk stratification or prevention.

A clear understanding of which patients are at the highest risk for splenic infarction is crucial. Identifying specific anatomical factors (e.g., spleen size, shunt characteristics) and procedural factors (e.g., glue volume) would allow for better pre-procedural risk assessment, informed patient consent, and potentially, modification of technical approaches (e.g., controlled glue injection, coil-assisted embolization of shunts) in high-risk scenarios (19). Therefore, we conducted this study with the primary aims to: (1) determine the proportion of splenic infarction among patients with available post-procedural computed tomography (CT) after endoscopic glue injection for GV, with particular attention to its association with repeated injections; (2) explore patient-related, anatomical, and procedural correlates of splenic infarction; and (3) describe its clinical presentation, management, and short-term outcomes.

## 2 Methods

### 2.1 Study design and patient population

This single-center, retrospective, observational cohort study was conducted at The Fifth People's Hospital of Ganzhou City between January 2018 and February 2025. The study protocol was reviewed and approved by the Institutional Review Board of The Fifth People's Hospital of Ganzhou City, which waived the requirement for informed consent due to the retrospective nature of the study.

We screened the electronic medical records of all consecutive adult patients (aged ≥18 years) with liver cirrhosis who underwent endoscopic cyanoacrylate glue injection for the management of GV, either for acute bleeding or primary/secondary prophylaxis. The diagnosis of cirrhosis was based on established clinical, laboratory, radiological, or histological criteria (20). The key exclusion criteria were: (1) absence of post-procedural CT imaging available for review in our hospital's system; (2) pre-existing splenic infarction prior to the endoscopic procedure; and (3) concomitant conditions that could predispose to splenic infarction (e.g., atrial fibrillation, infective endocarditis, hematological malignancies, or recent trauma).

**Figure 1:**
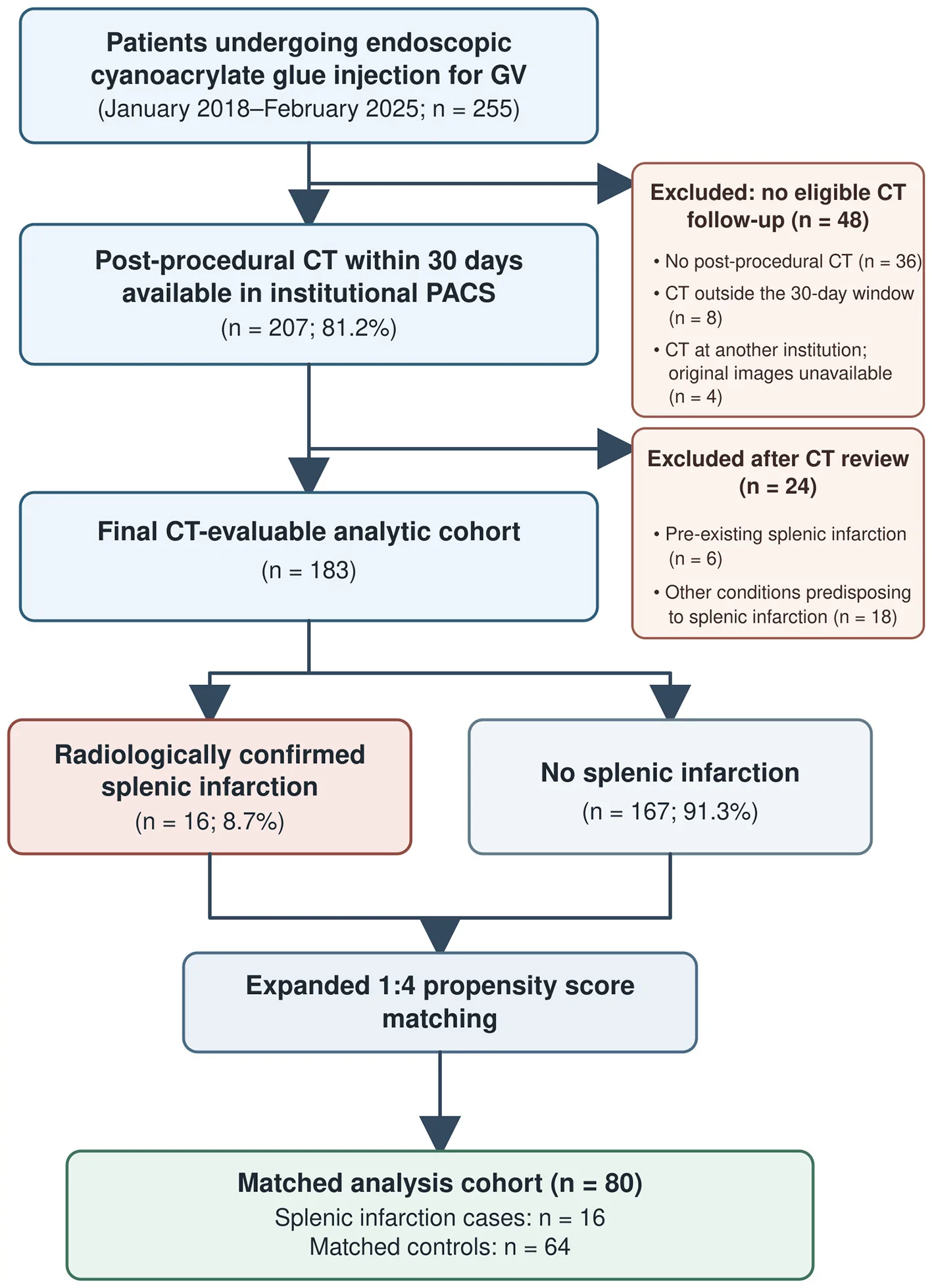
Flowchart of patient selection and propensity score matching. GV, gastric varices; CT, computed tomography; PACS, picture archiving and communication system.

During the study period, 255 unique patients underwent at least one glue injection procedure. Of these, 207 patients (81.2%) underwent post-procedural CT within 30 days and had images available for review in the institutional Picture Archiving and Communication System (PACS). Among the remaining 48 patients without eligible CT follow-up, 36 did not undergo CT, eight underwent CT outside the prespecified 30-day window, and four underwent CT at another institution but the original images were unavailable for review. Among the 207 patients with evaluable CT, six were excluded because of pre-existing splenic infarction and 18 because of concomitant conditions that could independently predispose to splenic infarction. Consequently, 183 patients constituted the final CT-evaluable analytic cohort (Figure 1).

To examine possible re-exposure after a recognized infarction, the records of the entire 255-patientsource cohort, including the six patients excluded for pre-existing splenic infarction, were additionally reviewed for the documented temporal relationship between prior glue-injection sessions and infarction and for any subsequent glue injection after recognition of the infarction.

### 2.2 Endoscopic procedure and data collection

All endoscopic procedures were performed by experienced endoscopists at our institution using standard video gastroscopes. All patients were treated with the same N-butyl-2-cyanoacrylate tissue adhesive using a standardized sandwich injection technique. Briefly, the injection catheter was first flushed with 3 ml of 5% glucose solution. N-butyl-2-cyanoacrylate was then mixed with lipiodol at a fixed ratio of 1:1.5 and injected into the gastric varix, followed immediately by another flush of 5% glucose solution. This sandwich technique was used to reduce premature polymerization within the catheter and to limit excessively rapid glue migration.

Data were meticulously extracted from our hospital's electronic medical records using a standardized data collection form. The collected data included: (1) patient demographics and baseline characteristics, including age, sex, and etiology of cirrhosis; (2) liver disease severity, including Child-Pugh class, MELD-Na score, platelet count, albumin, and total bilirubin obtained within one week before the procedure; (3) treatment history, including the number of previous endoscopic glue injection sessions for GV; (4) pre-index endoscopic characteristics, including maximum GV diameter, size category, morphology, Sarin classification, and index-session variceal status; (5) pre-procedural imaging parameters, including splenic longitudinal diameter, SPSS type and maximum diameter, and portal vein thrombosis; (6) treatment indication; and (7) index-session procedural details, including glue volume, number of injection sites, and adjunctive sclerotherapy.

Pre-index endoscopic characteristics were obtained from the index-session endoscopy reports and, when available, verified using archived endoscopic images. Maximum GV diameter was analyzed continuously and categorized as < 10 mm, 10–20 mm, or >20 mm. Morphology was classified as simple nodular or complex, with complex morphology including clustered, tortuous, or conglomerate/tumorous varices. GV were classified as GOV1, GOV2, IGV1, or IGV2 according to the Sarin classification (21). Previously untreated/*de novo* varices referred to target varices without previous treatment. Residual varices were defined as persistent varices after previous therapy without documented complete obliteration, whereas recurrent varices were defined as varices that reappeared after previously documented obliteration. Cardiofundal varices comprised GOV2 and IGV1.

For the purpose of this analysis, a history of ≥2 previous glue injection sessions was defined as two or more prior sessions for GV, regardless of acute bleeding or prophylaxis. The median interval between the previous injection and the index procedure was 4.5 months (IQR 2.8–7.3 months), with all repeat procedures performed at least 1 month after the prior session to allow for assessment of treatment response. For patients with a history of multiple glue injection sessions, the first session with available post-procedural CT follow-up during the study period was defined as the index session and included in the analysis to ensure independence of observations. This index session did not necessarily represent the patient's first-ever glue injection session. The glue volume recorded for analysis referred only to the amount of cyanoacrylate administered during this index session, rather than the cumulative glue volume across all previous treatment sessions. The number of prior glue injection sessions before the index session was recorded separately as a key treatment-history exposure variable.

### 2.3 Outcome definition and assessment

The primary outcome was radiologically confirmed splenic infarction within 30 days after endoscopic glue injection among CT-evaluable patients. Splenic infarction was defined as a new wedge-shaped or geographic hypodense area in the spleen on post-procedural CT compared with available pre-procedural imaging. Post-procedural CT was obtained at the discretion of the treating clinical team. Indications included clinical suspicion of a post-procedural complication, such as left upper quadrant pain, fever, unexplained leukocytosis, or other clinical deterioration, as well as selective follow-up in patients considered to be at higher procedural risk. The primary indication for CT was retrospectively classified as clinical suspicion, selective follow-up, or unclear. The indications and timing of CT were not governed by a uniformly applied protocol and could vary among clinicians and across the study period. Consequently, the present study estimates the proportion of splenic infarction among CT-evaluable patients rather than its incidence among the entire treated population. All relevant CT images stored in the PACS were independently re-evaluated by two experienced abdominal radiologists who were blinded to the clinical data.

Any discrepancies in interpretation were resolved by consensus with a third senior radiologist. The infarct extent was semi-quantitatively estimated as a percentage of total splenic volume (< 30%, minor; 30%−50%, moderate; >50%, major). Secondary outcomes included factors associated with splenic infarction, its clinical management, and short-term clinical outcomes such as 30-day mortality, rebleeding, other complications, and length of hospital stay.

### 2.4 Propensity score matching

Given the significant imbalance in group sizes between the infarction (*n* = 16) and non-infarction (*n* = 167) cohorts, an expanded 1:4 propensity score matching (PSM) approach was used to reduce measured baseline imbalances within the CT-evaluable cohort. The propensity score, representing the probability of developing splenic infarction, was calculated for each patient using a multivariable logistic regression model that included the following pre-specified covariates: age, sex, Child-Pugh class (categorized as A, B, or C), MELD-Na score, platelet count, albumin, total bilirubin, and index-session endoscopic characteristics. Nearest-neighbor matching without replacement was employed with a caliper width of 0.02 of the standard deviation of the logit of the propensity score to ensure close matching. Covariate balance was assessed using standardized mean differences (SMD), with SMD no greater than 0.10 prespecified as acceptable balance. For variables with more than two categories, an overall generalized SMD was reported.

### 2.5 Statistical analysis

Statistical analyses were performed using SPSS version 26.0 (IBM Corp., Armonk, NY, USA). Continuous variables were assessed for distributional normality. Normally distributed continuous variables are presented as mean ± standard deviation and were compared using the independent-samples *t-*test, whereas non-normally distributed continuous variables are presented as median with interquartile range and were compared using the Mann–Whitney *U* test. Categorical variables are presented as frequencies and percentages and were compared using Pearson's chi-square test or Fisher's exact test, as appropriate.

Univariate Firth penalized logistic regression was first performed for each candidate variable. Two multivariable Firth penalized logistic regression models were then constructed. Because all infarction cases had a history of ≥2 previous injections, complete separation occurred; the Firth estimate for this exposure was retained for model transparency but was not interpreted as a stable quantitative effect size. Results are expressed as odds ratios (OR) or adjusted odds ratios (aOR) with 95% confidence intervals (CI). A two-sided *p*-value < 0.05 was considered statistically significant.

## 3 Results

### 3.1 Patient characteristics and PSM

A total of 255 unique adult patients underwent endoscopic glue injection for GV. Post-procedural CT within 30 days was available for 207 patients (81.2%), whereas 48 patients had no eligible CT follow-up. After excluding six patients with pre-existing splenic infarction and 18 with alternative conditions predisposing to splenic infarction, 183 patients constituted the final CT-evaluable analytic cohort. Among these 183 patients, the primary indications for post-procedural CT were clinical suspicion in 112 patients (61.2%), selective follow-up in 58 patients (31.7%), and unclear in 13 patients (7.1%). Splenic infarction was radiologically confirmed in 16 patients, corresponding to a proportion of 8.7% (16/183) among CT-evaluable patients. All 16 patients with splenic infarction had a history of ≥2 previous glue injection sessions, compared with 28 of 167 patients (16.8%) without infarction. Expanded 1:4 PSM was subsequently performed using demographic, liver-disease, laboratory, and pre-index endoscopic variables.

Review of the entire 255-patient source cohort, including the six patients excluded for pre-existing splenic infarction, showed that none underwent another glue-injection session after the infarction had been clinically or radiologically recognized, irrespective of its presumed cause. Because imaging was not routinely performed after every earlier session, repeat treatment after an unrecognized asymptomatic infarction cannot be excluded.

Before matching, patients with splenic infarction had more advanced liver disease, a greater recorded index-session GV burden, larger size category, more complex morphology, more cardiofundal varices, and more residual or recurrent varices (overall SMD = 0.800–1.207), indicating substantial potential confounding. After expanded PSM, 64 controls were matched to the 16 infarction cases, and matching substantially reduced the measured differences. However, platelet count (SMD = 0.113), albumin (SMD = 0.119), and the four-category Sarin classification (generalized SMD = 0.179) remained above the prespecified 0.10 threshold, indicating residual imbalance. All other covariates included in matching had SMD values no greater than 0.10. History of ≥ 2 previous injections was intentionally excluded from matching because it was the principal exposure and remained markedly imbalanced (100.0% vs. 15.6%; SMD = 3.286; Table 1).

**Table 1:** Baseline characteristics and index-session endoscopic findings before and after expanded PSM.

Characteristic	Before matching			After matching		
	Splenic infarction (*n* = 16)	No infarction (*n* = 167)	SMD	Splenic infarction (*n* = 16)	No infarction (*n* = 64)	SMD
Demographics						
Age, years, Mean ± SD	58.8 ± 8.5	55.9 ± 10.1	0.311	58.8 ± 8.5	58.1 ± 8.9	0.079
Male, *n* (%)	12 (75.0)	116 (69.5)	0.124	12 (75.0)	46 (71.9)	0.071
Liver function severity						
Child-pugh class, *n* (%)			0.486			0.080
A	3 (18.8)	67 (40.1)		3 (18.8)	14 (21.9)	
B	10 (62.5)	74 (44.3)		10 (62.5)	38 (59.4)	
C	3 (18.8)	26 (15.6)		3 (18.8)	12 (18.8)	
MELD-Na score, median (IQR)	18 (15–23)	14 (10–19)	0.645	18 (15–23)	17.5 (14–22)	0.092
Laboratory parameters, Mean ±SD						
Platelet count (×1010^9^/L)	55.0 ± 21.8	69.1 ± 28.7	0.553	55.0 ± 21.8	57.5 ± 22.4	0.113
Albumin (g/L)	28.5 ± 5.1	30.9 ± 5.2	0.466	28.5 ± 5.1	29.1 ± 5.0	0.119
Total bilirubin (μmol/L)	46.8 ± 21.5	38.5 ± 18.7	0.412	46.8 ± 21.5	45.2 ± 19.9	0.077
Index-session endoscopic characteristics						
Maximum GV diameter, mm, mean ± SD	23.6 ± 6.4	17.0 ± 6.1	1.056	23.6 ± 6.4	23.1 ± 6.2	0.079
Varix size category, *n* (%)			0.800			0.000
<10 mm	1 (6.3)	32 (19.2)		1 (6.3)	4 (6.3)	
10–20 mm	5 (31.3)	91 (54.5)		5 (31.3)	20 (31.3)	
>20 mm	10 (62.5)	44 (26.3)		10 (62.5)	40 (62.5)	
Complex morphology, *n* (%)	12 (75.0)	55 (32.9)	0.931	12 (75.0)	48 (75.0)	0.000
Sarin classification, *n* (%)			0.871			0.179
GOV1	1 (6.3)	58 (34.7)		1 (6.3)	4 (6.3)	
GOV2	9 (56.3)	58 (34.7)		9 (56.3)	36 (56.3)	
IGV1	6 (37.5)	43 (25.7)		6 (37.5)	23 (35.9)	
IGV2	0 (0.0)	8 (4.8)		0 (0.0)	1 (1.6)	
Index-session variceal status, *n* (%)			1.207			0.095
Previously untreated/de novo	3 (18.8)	117 (70.1)		3 (18.8)	14 (21.9)	
Residual varices	9 (56.3)	36 (21.6)		9 (56.3)	36 (56.3)	
Recurrent varices	4 (25.0)	14 (8.4)		4 (25.0)	14 (21.9)	
Treatment history						
≥2 Previous injections, *n* (%)	16 (100.0)	28 (16.8)	3.151	16 (100.0)	10 (15.6)	3.286

### 3.2 Representative endoscopic and radiological findings

**Figure 2:**
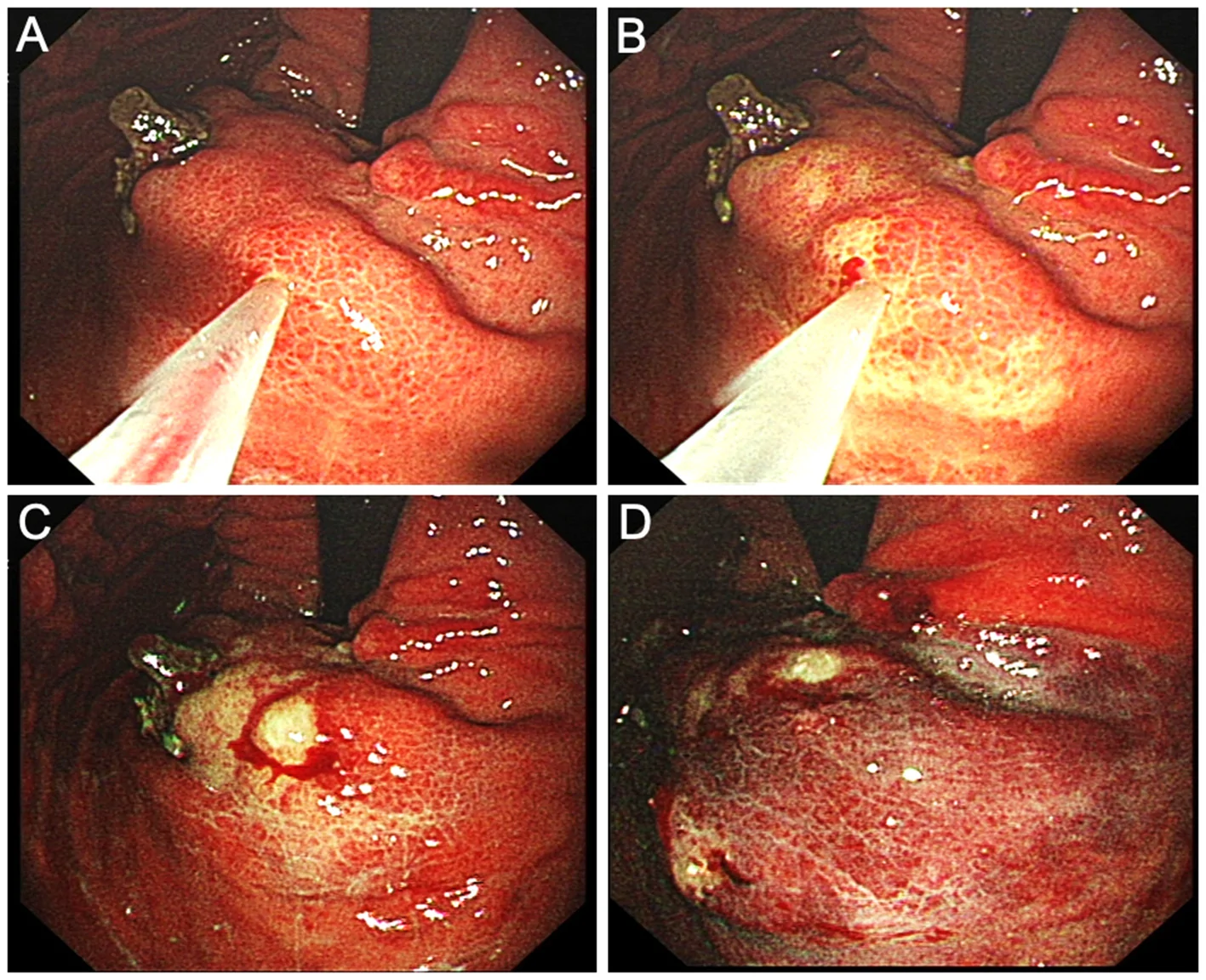
Representative endoscopic findings of cyanoacrylate glue injection for GV. **(A)** Injection needle inserted into the varix with visible blood return. **(B)** Mucosal whitening during glue injection. **(C)** Post-injection appearance after needle withdrawal, showing no obvious residual mucosal bulging around the injection site. **(D)** Progressive purple discoloration of the surrounding mucosa after injection.

Figure 2 demonstrates the key endoscopic steps and immediate mucosal changes during cyanoacrylate glue injection for GV. In detail, the injection needle was inserted directly into the GV, and visible blood return after aspiration confirmed intravariceal needle placement (Figure 2A). During cyanoacrylate injection, transient whitening of the surrounding gastric mucosa was observed (Figure 2B). After needle withdrawal, the previously elevated variceal area showed no obvious residual mucosal bulging around the injection site, suggesting effective intravariceal delivery rather than ineffective submucosal injection and early purple discoloration of the adjacent mucosa was also noted (Figure 2C). Shortly after injection, the surrounding mucosa gradually became more deeply and extensively purple, reflecting progressive local post-injection vascular and mucosal changes (Figure 2D).

**Figure 3:**
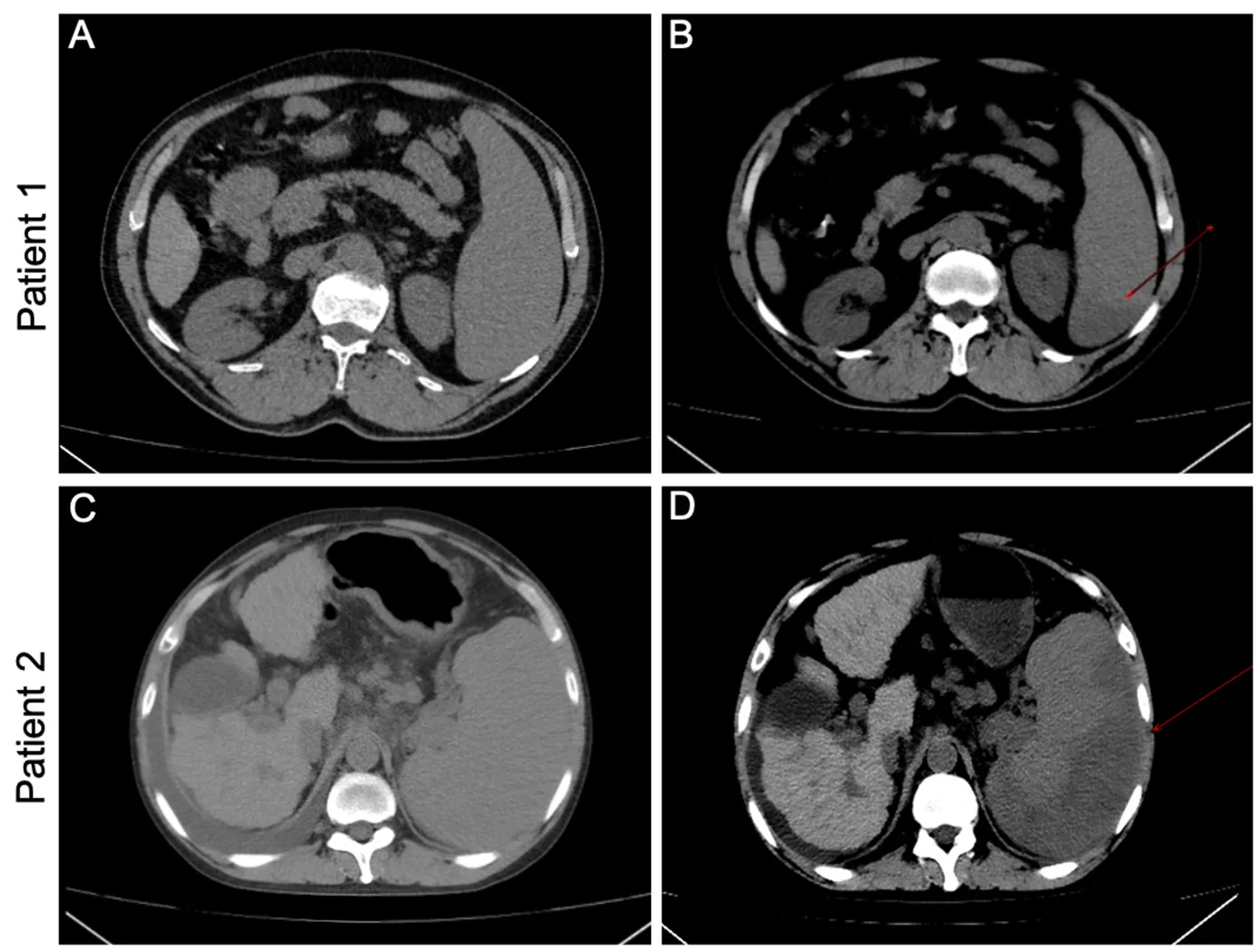
Representative CT images of splenic infarction before and after endoscopic glue injection. **(A)** Pre-procedural CT of Patient 1 showing mild splenomegaly. **(B)** Post-procedural CT of Patient 1 demonstrating a wedge-shaped peripheral hypodense infarct (red arrow). **(C)** Pre-procedural CT of Patient 2 showing marked splenomegaly. **(D)** Post-procedural non-contrast CT of Patient 2 revealing a large geographic hypodense infarct involving approximately 50% of the splenic volume (red arrow), with minimal peri-splenic fluid.

Figure 3 presents representative CT images from two patients who developed splenic infarction following endoscopic glue injection. The pre-procedural CT (Figure 3A) in patient 1 shows mild splenic abnormalities. However, the post-procedural CT (Figure 3B) reveals a wedge-shaped peripheral hypodense area in the spleen (red arrow), which represents the most common imaging pattern of splenic infarction, observed in 75.0% of cases in our cohort. The pre-procedural CT (Figure 3C) in patient 2 demonstrates marked splenomegaly. The post-procedural non-contrast CT (Figure 3D) shows an extensive geographic hypodense infarct involving approximately 50% of the splenic volume, with minimal peri-splenic fluid. The infarct is well-demarcated and clearly visible even without contrast administration. This case exemplifies the potential for extensive infarction in patients with pronounced splenomegaly, an exploratory clinical marker in this cohort.

**Figure 4:**
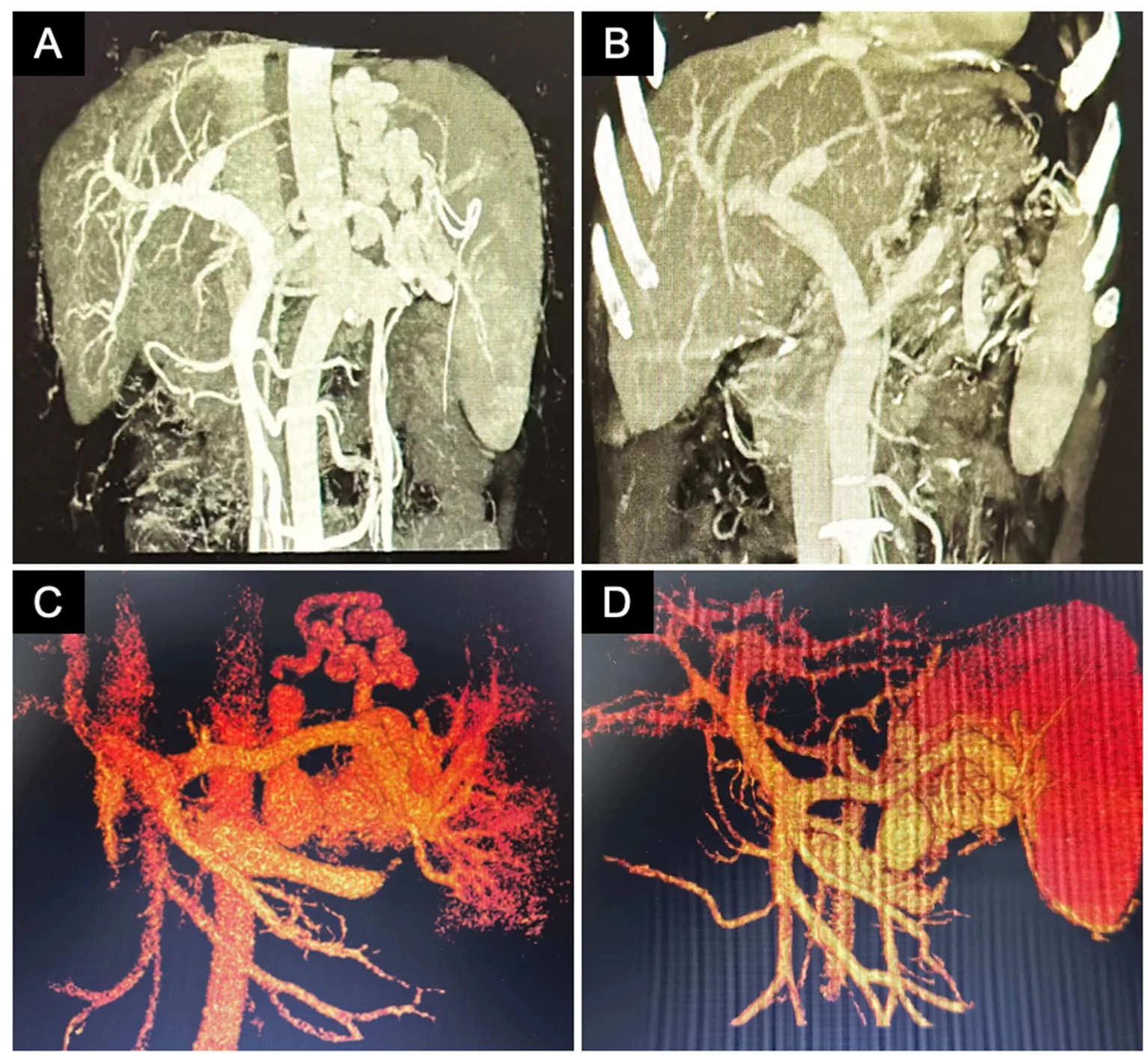
Representative imaging changes in large spontaneous portosystemic shunts before and after endoscopic cyanoacrylate injection. **(A) and (B)** Coronal CT angiographic images of a large gastrorenal shunt before **(A)** and after **(B)** endoscopic cyanoacrylate injection. **(C) and (D)** Three-dimensional vascular reconstruction images of a large splenorenal shunt before **(C)** and after **(D)** glue injection.

Figure 4 shows representative imaging changes in large SPSS (≥10 mm) before and after endoscopic cyanoacrylate injection. Figures 4A, B show coronal CT angiographic images of a large gastrorenal shunt before and after glue injection. After treatment, the GV complex and its associated gastrorenal collateral pathway showed interval morphological changes, with altered vascular filling and partial reduction of the variceal/shunt complex. Figures 4C, D show three-dimensional vascular reconstruction images of a large splenorenal shunt before and after glue injection, demonstrating post-procedural changes in the reconstructed collateral network adjacent to the spleen. Although the actual cyanoacrylate migration path was not directly visible, these pre- and post-procedural images demonstrate that large SPSS may undergo appreciable anatomical and hemodynamic changes after glue injection.

### 3.3 Comparison of endoscopic, anatomical, and procedural characteristics in the matched cohort

In the matched cohort, the recorded index-session endoscopic characteristics were comparable between groups. Maximum GV diameter was 23.6 ± 6.4 mm in the infarction group and 23.1 ± 6.2 mm in the non-infarction group (*p* = 0.781). The proportions with a maximum GV diameter >20 mm were identical (62.5% vs. 62.5%, *p* = 1.000), as were the proportions with complex morphology (75.0% vs. 75.0%, *p* = 1.000). Cardiofundal varices were present in 93.8% vs. 92.2% (*p* = 1.000), and residual or recurrent varices were present in 81.3% vs. 78.1% (*p* = 1.000), respectively.

As established, all patients in the infarction group had a history of multiple glue injections. Patients with splenic infarction had significantly larger splenic longitudinal diameters (17.5 ± 2.1 cm vs. 15.6 ± 2.2 cm, *p* = 0.003) and a markedly higher prevalence of SPSS (87.5% vs. 45.3%, *p* = 0.002). Among those with shunts, gastrorenal shunts were the most common type in both groups, but the shunt diameter was substantially larger in the infarction group (median 12.0 mm vs. 7.0 mm, *p* < 0.001). Furthermore, 81.3% of infarction patients had a large shunt (diameter ≥10 mm) compared to only 21.9% in the control group (*p* < 0.001).

Despite the comparable measured index-session GV burden, index-session glue volume was significantly higher in the infarction group (2.8 ± 0.9 ml vs. 2.0 ± 0.6 ml, *p* < 0.001), and a volume >2.5 ml was more frequently administered (62.5% vs. 20.3%, *p* < 0.001). In contrast, there were no significant differences in treatment indication (emergent, 68.8% vs. 62.5%, *p* = 0.642), number of injection sites [median 2 (IQR 2–2) vs. 2 (IQR 1–2), *p* = 0.102], or adjunctive sclerotherapy (37.5% vs. 31.3%, *p* = 0.633) during the index session (Table 2). The matched comparison showed a difference in glue volume despite similar measured maximum GV diameter; however, residual confounding by unmeasured vascular and procedural complexity cannot be excluded.

**Table 2:** Index-session endoscopic, anatomical, and procedural characteristics in the PSM cohort (*n* = 80).

Variable	Splenic infarction (*n* = 16)	No infarction (*n* = 64)	*P*-value
Index-session endoscopic characteristics			
Maximum GV diameter (mm), Mean ± SD	23.6 ± 6.4	23.1 ± 6.2	0.781
Maximum GV diameter >20 mm, *n* (%)	10 (62.5)	40 (62.5)	1.000
Complex morphology, *n* (%)	12 (75.0)	48 (75.0)	1.000
Cardiofundal varices (GOV2/IGV1), *n* (%)	15 (93.8)	59 (92.2)	1.000
Residual or recurrent varices, *n* (%)	13 (81.3)	50 (78.1)	1.000
Preprocedural anatomical factors			
Splenic longitudinal diameter (cm), Mean ± SD	17.5 ± 2.1	15.6 ± 2.2	0.003
Spontaneous portosystemic shunt			
Presence of any shunt, *n* (%)	14 (87.5)	29 (45.3)	0.002
Shunt Type, *n* (%)			0.005
Gastrorenal shunt	11 (68.8)	26 (40.6)	
Splenorenal shunt	3 (18.8)	3 (4.7)	
Shunt diameter (mm), Median (IQR)	12.0 (10.3–14.8)	7.0 (5.0–9.0)	<0.001
Shunt diameter ≥ 10 mm, *n* (%)	13 (81.3)	14 (21.9)	<0.001
Presence of portal vein thrombosis, *n* (%)	5 (31.3)	17 (26.6)	0.707
Procedure details			
Treatment indication (Emergent), *n* (%)	11 (68.8)	40 (62.5)	0.642
Glue injection			
Index-session glue volume (ml), Mean ± SD	2.8 ± 0.9	2.0 ± 0.6	<0.001
Index-session glue volume > 2.5 ml, *n* (%)	10 (62.5)	13 (20.3)	<0.001
Number of injection sites, Median (IQR)	2 (2–2)	2 (1–2)	0.102
Adjunctive sclerotherapy, *n* (%)	6 (37.5)	20 (31.3)	0.633

### 3.4 Exploratory firth regression analysis

All patients who developed splenic infarction had a history of ≥2 previous glue injection sessions; therefore, complete separation was observed for this exposure. Firth penalized logistic regression was applied to reduce small-sample bias and obtain finite estimates. However, Firth penalization does not restore the information absent from the zero cell, and this estimate should not be interpreted as a stable quantitative effect size. In univariate analysis, history of ≥2 previous injections, larger splenic diameter, large SPSS, and higher index-session glue volume showed statistical signals in exploratory analyses (*p* < 0.05). In contrast, maximum GV diameter was not significant when analyzed per 5 mm (OR 1.07, 95% CI 0.74–1.56; *p* = 0.724) or as >20 mm (OR 1.00, 95% CI 0.29–3.41; *p* = 1.000).

After maximum GV diameter was retained as a separate covariate, the association between index-session glue volume and splenic infarction was attenuated but continued to show a nominal statistical signal in the exploratory model. For history of ≥2 previous injections, Model 1 yielded a finite Firth estimate (aOR 31.82, 95% CI 2.55–397.03; *p* = 0.007) that is reported only for transparency and is not interpreted quantitatively. Exploratory Model 1 estimates were also observed for larger splenic diameter (per 1 cm: aOR 1.39, 95% CI 1.01–1.92; *p* = 0.045), large SPSS (aOR 4.02, 95% CI 1.07–15.08; *p* = 0.040), and higher index-session glue volume (per 1 ml: aOR 2.05, 95% CI 1.03–4.08; *p* = 0.041). Maximum GV diameter did not show a statistical signal in the exploratory adjusted model with infarction (per 5 mm: aOR 1.08, 95% CI 0.72–1.62; *p* = 0.714). Model 2 similarly yielded a finite but unstable estimate for history of ≥2 previous injections (aOR 29.47, 95% CI 2.43–357.43; *p* = 0.008), while exploratory estimates were also observed for splenic diameter >16 cm (aOR 4.52, 95% CI 1.13–18.09; *p* = 0.033), large SPSS (aOR 4.13, 95% CI 1.10–15.54; *p* = 0.036), and index-session glue volume >2.5 ml (aOR 3.49, 95% CI 1.01–12.08; *p* = 0.048). Maximum GV diameter >20 mm was not independently associated with infarction (aOR 1.18, 95% CI 0.30–4.60; *p* = 0.812; Table 3).

**Table 3:** Exploratory bias-reduced firth regression estimates in the PSM cohort.

Variable	Univariate Firth [OR (95% CI)]	*P*-value	Multivariable Model 1 [aOR (95% CI)]	*P*-value	Multivariable Model 2 [aOR (95% CI)]	*P*-value
History of ≥2 previous injections (Yes vs. No)	171.29 (9.52–3082.15)	<0.001	31.82 (2.55–397.03)	0.007	29.47 (2.43–357.43)	0.008
Splenic diameter (per 1 cm)	1.55 (1.15–2.08)	0.004	1.39 (1.01–1.92)	0.045		
Splenic diameter >16 cm	6.92 (1.96–24.41)	0.003			4.52 (1.13–18.09)	0.033
Large SPSS ≥10 mm	13.43 (3.61–49.92)	<0.001	4.02 (1.07–15.08)	0.040	4.13 (1.10–15.54)	0.036
Maximum GV diameter, per 5 mm	1.07 (0.74–1.56)	0.724	1.08 (0.72–1.62)	0.714		
Maximum GV diameter >20 mm	1.00 (0.29–3.41)	1.000			1.18 (0.30–4.60)	0.812
Index-session glue volume, per 1 ml	2.96 (1.51–5.79)	0.002	2.05 (1.03–4.08)	0.041		
Index-session glue volume >2.5 ml	6.16 (1.96–19.41)	0.002			3.49 (1.01–12.08)	0.048

Complete separation occurred for history of ≥2 previous injections because all 16 infarction cases had this exposure. The finite Firth estimates are presented solely for model transparency and should not be interpreted as stable quantitative effect sizes. All other estimates are exploratory owing to the limited number of events and wide confidence intervals.

### 3.5 Clinical presentation and course of splenic infarction

The clinical details of the 16 patients with splenic infarction are summarized in Table 4. The infarction was diagnosed at a median of 2 days post-procedure (IQR 1–4). The majority of patients (81.3%) were symptomatic, primarily presenting with left upper quadrant pain (68.8%) and fever (50.0%). The median infarct extent was 35% of the splenic volume, with wedge-shaped, peripheral infarcts being the most common pattern (75.0%).

**Table 4:** Detailed clinical course, management, and outcomes of patients with splenic infarction (*n* = 16).

Characteristic	Result
Presentation & diagnosis	
Time to diagnosis (post-procedure), days, Median (IQR)	2 (1–4)
Symptomatic at diagnosis, *n*(%)	13 (81.3)
Left Upper Quadrant Pain, *n*(%)	11 (68.8)
Fever, *n*(%)	8 (50.0)
Peak Body Temperature (°C), Mean ± SD	38.3 ± 0.7
Peak CRP (mg/L), Median (IQR)	80 (40–120)
Infarct Characteristics on CT	
Infarct Extent (% of splenic volume), Median (IQR)	35 (20–50)
Infarct pattern, *n* (%)	
Wedge-shaped, peripheral	12 (75.0)
Global/Massive	3 (18.8)
Multiple, patchy	1 (6.3)
Presence of peri-splenic fluid, *n* (%)	3 (18.8)
Management	
Conservative (analgesia + monitoring)	15 (93.8)
Antibiotic therapy initiated	3 (18.8)
Percutaneous drainage	1 (6.3)
Splenectomy	0 (0)
Clinical course	
Time to symptomatic resolution (days), Median (IQR)	7 (5–9)
Follow-up imaging evolution (*n* = 14 with follow-up CT)	
Complete resolution	2 (14.3%)
Partial resolution (scarring)	10 (71.4%)
No change	1 (7.1%)
Progression to abscess	1 (7.1%)

Management was predominantly conservative (93.8%), with antibiotics initiated in 18.8% of cases for suspected infection. One patient required percutaneous drainage. The median time to symptomatic resolution was 7 days. Follow-up imaging available for 14 patients showed that most infarcts evolved into partial resolution with scarring (71.4%), while one case (7.1%) progressed to a splenic abscess.

### 3.6 Short-term clinical outcomes

Comparison of short-term outcomes in the matched cohort (*n* = 80) showed that the 30-day all-cause mortality and variceal rebleeding rates were not significantly different between the splenic infarction and non-infarction groups. However, the development of splenic infarction was associated with a significantly longer median hospital stay (14 days vs. 10 days, *p* = 0.008). There were no significant differences in the rates of ICU admission or other infection-related complications between the groups (Table 5).

**Table 5:** Short-term clinical outcomes in the matched cohort (*n* = 80).

Outcome	Splenic infarction (*n* = 16)	No infarction (*n* = 64)	*P*-value
Mortality & morbidity			
30-day All-cause Mortality, *n* (%)	1 (6.3)	2 (3.1)	0.493
Variceal Rebleeding, *n* (%)	2 (12.5)	7 (10.9)	1.000
Infection-related complications			
Any documented infection, *n* (%)	5 (31.3)	15 (23.4)	0.519
Spontaneous bacterial peritonitis	2 (12.5)	5 (7.8)	0.622
Pneumonia	1 (6.3)	5 (7.8)	1.000
Resource utilization			
ICU Admission required, *n* (%)	3 (18.8)	5 (7.8)	0.194
Hospital stay, days, Median (IQR)	14 (10–20)	10 (7–14)	0.008

## 4 Discussion

This single-center retrospective study provides a comprehensive analysis of splenic infarction, a notable yet under-investigated complication following endoscopic cyanoacrylate glue injection for GV. The principal findings can be summarized as follows: First, splenic infarction was identified in 8.7% of patients in our CT-evaluable cohort, which is substantially higher than previously perceived from isolated case reports (22–24). Notably, this figure represents the proportion of radiologically confirmed splenic infarction among patients with available post-procedural CT and should not be interpreted as the true incidence among all patients undergoing endoscopic glue injection. Nevertheless, the observed rate suggests that splenic infarction may be under-recognized, particularly when post-procedural imaging is not systematically performed. Second, exploratory models showed statistical signals for repeated injections, larger splenic diameter, large SPSS, and higher index-session glue volume, but these variables should not be interpreted as independent or actionable risk factors. Third, while the complication was managed conservatively in most cases and no statistically detectable difference in short-term mortality was observed, it led to a significantly prolonged hospital stay, underscoring its clinical and economic impact.

The observed proportion of 8.7% should be interpreted strictly within the selected CT-evaluable cohort. Post-procedural CT was frequently prompted by clinical symptoms or selectively obtained in patients considered to be at higher procedural risk, which may have enriched the analytic cohort for patients with a greater probability of splenic infarction. Conversely, asymptomatic infarctions among patients who did not undergo CT could not be detected. Because these competing selection mechanisms may have operated simultaneously, neither the magnitude nor the direction of the difference between the observed 8.7% and the proportion among all treated patients can be determined reliably. Previous reports were mainly isolated case reports without a defined imaging denominator, making direct numerical comparison inappropriate (25). The population-level proportion therefore remains unknown and should be evaluated prospectively using predefined and standardized post-procedural imaging.

The same imaging-selection mechanism may also have influenced the observed associations. Patients with repeated procedures, marked splenomegaly, large shunts, or higher glue volumes may have been more likely to undergo post-procedural CT because they were perceived to be at greater procedural risk. Conditioning cohort entry on CT availability could therefore distort the estimated associations. Although PSM and Firth penalized regression reduced measured confounding within the CT-evaluable cohort, neither method could eliminate selection and surveillance bias occurring before cohort inclusion. Accordingly, these findings should be interpreted as associations within a selected population and require prospective validation before being used for causal inference or formal risk prediction. In addition, the unmatched endoscopic data support the concern that GV severity was an important confounder. Patients requiring repeated sessions may have had larger, more complex, cardiofundal, or residual/recurrent varices at the index procedure. After these recorded endoscopic features were incorporated into expanded PSM, history of ≥2 previous injections remained markedly imbalanced; however, all infarction cases had this exposure, producing complete separation, and the Firth estimate should not be interpreted as a stable quantitative effect size. Repeated treatment, splenomegaly, large SPSS, and higher glue volume may represent an interrelated phenotype of advanced portal hypertension, refractory varices, and treatment complexity rather than separate, actionable risk factors. Because portal pressure, portal and collateral flow, and longitudinal disease progression were not directly measured, residual confounding by disease severity remains.

The pathophysiological mechanisms underlying the identified associations are plausible and align with the hemodynamic alterations of portal hypertension (26). The present study identified associations of splenic infarction with a history of ≥2 previous glue-injection sessions, larger splenic diameter, large SPSS, and higher index-session glue volume. Previous case reports have documented splenic infarction, including splenic artery occlusion, after cyanoacrylate injection, confirming that this complication can occur; however, these reports do not establish a uniform embolic pathway (27). A radiological study of pulmonary glue embolism demonstrated migration of cyanoacrylate through gastrorenal or splenorenal shunts and reported an association with greater injected volume, although these findings concerned pulmonary rather than splenic embolization (28). Significant SPSS has also been regarded as high-risk anatomy for ectopic embolization in studies evaluating treatments for GV (29). Nevertheless, the present study did not directly demonstrate glue migration through SPSS or into the splenic arterial circulation. Portal pressure and flow, longitudinal changes in shunt diameter, and cumulative glue volume across previous sessions were not systematically measured. Therefore, altered portal hemodynamics, treatment-related changes in shunt anatomy, and cumulative glue exposure should be regarded as mechanistic hypotheses rather than mechanisms established by our data, and the observed associations should not be interpreted as evidence of causality.

Index-session glue volume was clinically size-informed and was therefore potentially confounded by GV burden. However, no fixed size-based dosing formula was used. After maximum GV diameter, complex morphology, cardiofundal location, and residual or recurrent status were addressed through expanded PSM, and maximum GV diameter was entered separately into both Firth models, glue volume remained associated with splenic infarction, whereas maximum diameter itself was not independently associated. This finding suggests that glue volume may capture procedural features beyond maximum diameter alone, including unmeasured communicating channels, intravariceal flow, the extent of the treated vascular bed, or technical difficulty (30). Because these characteristics were not fully quantified, the residual association should not be interpreted as proof of a causal dose-response relationship or as validation of a universal glue-volume threshold.

The clinical course observed in our cohort offers valuable insights for management. The predominance of abdominal pain and fever as presenting symptoms is consistent with the typical presentation of splenic infarction (31). Importantly, the majority of patients (93.8%) were successfully managed with conservative measures, including analgesia and monitoring, indicating that non-operative management is sufficient in most cases. However, the progression to splenic abscess in one patient (6.3%) serves as a critical reminder to vigilantly monitor for signs of secondary infection, which may necessitate invasive intervention (32). The most significant impact on patient outcome was the prolonged hospitalization (median 14 vs. 10 days). The additional days are likely attributable to pain control, management of fever, and exclusion of other complications, thereby increasing healthcare utilization and costs.

These exploratory findings alone should not determine retreatment, glue-volume thresholds, or selection of alternative procedures. In patients being considered for retreatment, established clinical indications and vascular anatomy should continue to guide individualized multidisciplinary assessment and counseling. Alternative shunt-directed or coil-assisted approaches may be discussed when otherwise clinically appropriate (33), rather than selected on the basis of this exploratory model alone.

Several limitations should be acknowledged. First, the retrospective and single-center design introduced the possibility of unmeasured confounding despite adjustment using PSM. Matching substantially improved measured balance, but platelet count, albumin, and Sarin classification remained above the prespecified 0.10 SMD threshold. Second, only 207 of the 255 treated patients (81.2%) had evaluable post-procedural CT within 30 days, and the indications and timing of imaging were not uniformly standardized. CT was more likely to be obtained in symptomatic patients or those perceived to be at higher procedural risk, introducing selection and surveillance bias. Therefore, the observed 8.7% represents only the proportion among CT-evaluable patients and cannot be generalized to the entire treated population. Selection into the imaging cohort may also have influenced the estimated associations between procedural or anatomical factors and splenic infarction, and this bias could not be corrected by PSM or multivariable regression. Third, retrospective estimation of maximum GV diameter from endoscopy reports and archived images is less precise than standardized endoscopic ultrasonography. Residual confounding by communicating channels, intravariceal flow, wall tension, and operator judgment therefore remains possible. Repeated treatment, splenomegaly, and large shunts may also reflect unmeasured severity of portal hypertension. Finally, only 16 infarction events were available. Because all cases had a history of ≥2 previous injections, complete separation occurred; the corresponding Firth estimate is not a stable effect-size estimate despite penalization. The remaining multivariable estimates should also be considered exploratory because the number of events was limited and the confidence intervals were wide.

## 5 Conclusion

In conclusion, splenic infarction was radiologically confirmed in 16 of 183 CT-evaluable patients (8.7%) following endoscopic glue injection for GV. Within this selected cohort, repeated injections, larger splenic diameter, large SPSS, and higher index-session glue volume co-occurred with splenic infarction in exploratory analyses, but may collectively reflect more advanced portal hypertension and treatment complexity rather than separate, actionable risk factors. These findings may support heightened vigilance in patients requiring retreatment, but complete separation, residual imbalance, the small number of events, wide CI, imaging selection, and residual confounding preclude causal interpretation, precise effect-size estimation, or individual risk prediction. Prospective multicenter studies with standardized endoscopic and imaging assessment are needed to validate these exploratory findings.

## Data Availability

The original contributions presented in the study are included in the article/supplementary material, further inquiries can be directed to the corresponding author.

## References

[1] Sarin SK Lahoti D Saxena SP Murthy NS Makwana UK. Prevalence, classification and natural history of gastric varices: a long–term follow–up study in 568 portal hypertension patients. Hepatology. (1992) 16:1343–9. doi: 10.1002/hep.1840160607 1446890

[2] Castleberry DT Mann R Tharian B Thandassery RB. Endoscopic ultrasound in the management of complications related to cirrhosis- recent evidence. World J Gastrointest Endosc. (2025) 17:108549. doi: 10.4253/wjge.v17.i9.108549 40979066 PMC12444283

[3] Olevskaya ER Dolgushina AI Garbuzenko DV Khikhlova AO Saenko AA Khusainova GM . Role of endoscopy in the diagnosis and treatment of gastric mucosal lesions in liver cirrhosis patients. World J Gastrointest Endosc. (2025) 17:108787. doi: 10.4253/wjge.v17.i9.108787 40979065 PMC12444304

[4] Moctezuma-Velazquez C Abraldes JG. Future of endoscopy in surveillance of esophageal varices. Curr Gastroenterol Rep. (2025) 27:26. doi: 10.1007/s11894-025-00976-6 40156673

[5] Tanner S Schulman AR. Endoscopic management of portal hypertension and varices. Tech Vasc Interv Radiol. (2025) 28:101053. doi: 10.1016/j.tvir.2025.101053 40784688

[6] Karna R Are V Azeem N Trikudanathan G. Cyanoacrylate glue migration after endoscopic ultrasound guided embolization of peri-duodenal varices presenting as obstructive jaundice. Gastrointest Endosc. (2025) 103:370–1. doi: 10.1016/j.gie.2025.08.051 40914475

[7] Trikudanathan G Rahimi EF Bhatt A Bucobo JC Chandrasekhara V Copland AP . Endoscopic devices and techniques for the management of gastric varices (with videos). Gastrointest Endosc. (2025) 101:496–510. doi: 10.1016/j.gie.2024.06.038 39480369

[8] Ríos Castellanos E Seron P Gisbert JP Bonfill Cosp X. Endoscopic injection of cyanoacrylate glue versus other endoscopic procedures for acute bleeding gastric varices in people with portal hypertension. Cochrane Database Syst Rev. (2015) 2015:Cd010180. doi: 10.1002/14651858.CD010180.pub2 25966446 PMC10776035

[9] Plaz Torres MC Best LM Freeman SC Roberts D Cooper NJ Sutton AJ . Secondary prevention of variceal bleeding in adults with previous oesophageal variceal bleeding due to decompensated liver cirrhosis: a network meta-analysis. Cochrane Database Syst Rev. (2021) 3:Cd013122. doi: 10.1002/14651858.CD013122.pub2 33784794 PMC8094621

[10] Roccarina D Best LM Freeman SC Roberts D Cooper NJ Sutton AJ . Primary prevention of variceal bleeding in people with oesophageal varices due to liver cirrhosis: a network meta-analysis. Cochrane Database Syst Rev. (2021) 4:Cd013121. doi: 10.1002/14651858.CD013121.pub2 33822357 PMC8092414

[11] Hu Y Zhou M Liu D Gong J. Risk factors for glue extrusion bleeding after endoscopic injection of cyanoacrylate glue for gastric varices: a retrospective study of 269 patients. Dig Dis Sci. (2025) 70:2444–53. doi: 10.1007/s10620-025-08999-9 40164951

[12] Shi D Xu G Pan W. A randomized controlled trial comparing large-volume band ligator and cyanoacrylate injection in the endoscopic management of actively bleeding gastric varices. Sci Rep. (2025) 15:27134. doi: 10.1038/s41598-025-12600-8 40715299 PMC12297341

[13] Diaz Garcia G Boppana LKT Izzo C Ashby T Louis M. Pulmonary embolism: a complication of endoscopic variceal treatment. BMJ Case Rep. (2025) 18:e263011. doi: 10.1136/bcr-2024-263011 39842892

[14] Harth C De Mulder P Raevens S Ferdinande K Hindryckx P Geerts A . Fatal acute pulmonary embolism following endoscopic cyanoacrylate injection for gastric fundal varices. Acta Gastroenterol Belg. (2024) 87:531–4. doi: 10.51821/87.4.13020 39745042

[15] Manolakis A Tsagkidou K Koumarelas KE. Endoscopic ultrasound-guided therapies in the treatment of gastric varices: an in-depth examination of associated adverse events. World J Gastrointest Endosc. (2024) 16:640–6. doi: 10.4253/wjge.v16.i12.640 39735397 PMC11669960

[16] Samanta J Nabi Z Facciorusso A Dhar J Akbar W Das A . EUS-guided coil and glue injection versus endoscopic glue injection for gastric varices: international multicentre propensity-matched analysis. Liver Int. (2023) 43:1783–92. doi: 10.1111/liv.15630 37269164

[17] Khoury T Nadella D Wiles A Marshall C Kumar M Shapira G . A review article on gastric varices with focus on the emerging role of endoscopic ultrasound-guided angiotherapy. Eur J Gastroenterol Hepatol. (2018) 30:1411–5. doi: 10.1097/MEG.0000000000001200 29985209

[18] Wang X Yu S Chen X Duan L. Endoscopic ultrasound-guided injection of coils and cyanoacrylate glue for the treatment of gastric fundal varices with abnormal shunts: a series of case reports. J Int Med Res. (2019) 47:1802–9. doi: 10.1177/0300060519830207 PMC6460599 30819006

[19] Qin B Li A Liu S Zhuang K. Endoscopic ultrasound-guided selective inflow vessel devascularization: a novel approach in treatment of large gastric varices. Endoscopy. (2025) 57:E1080–e1. doi: 10.1055/a-2697-2542 PMC12445970 40967614

[20] Ginès P Krag A Abraldes JG Solà E Fabrellas N Kamath PS. Liver cirrhosis. Lancet. (2021) 398:1359–76. doi: 10.1016/S0140-6736(21)01374-X 34543610

[21] Luo X Hernández-Gea V. Update on the management of gastric varices. Liver Int. (2022) 42:1250–8. doi: 10.1111/liv.15181 35129288

[22] Köksal AS Kayaçetin E Torun S Erkan V Ökten RS. Splenic infarction after N-butyl-2-cyanoacrylate injection for gastric varices: why does it happen? Surg Laparosc Endosc Percutan Tech. (2013) 23:e191–3. doi: 10.1097/SLE.0b013e318272fd1e 24105294

[23] Qian SJ Huang ZY Tai Y Tang CW Wu H. Splenic infarction after sclerotherapy for gastric varices due to anatomical variation of left gastric artery: a case report and literature review. J Dig Dis. (2025) 26:88–91. doi: 10.1111/1751-2980.13334 40025778 PMC12038536

[24] Kurt M Onal IK Ibis M Tas A Ozderin YO Okten RS. Splenic infarction: rare complication of N-butyl-2-cyanoacrylate injection for gastric varices. Dig Endosc. (2010) 22:74–5. doi: 10.1111/j.1443-1661.2009.00922.x 20078672

[25] Sato T Yamazaki K Toyota J Karino Y Ohmura T Suga T. Gastric varices with splenic vein occlusion treated by splenic arterial embolization. J Gastroenterol. (2000) 35:290–5. doi: 10.1007/s005350050348 10777159

[26] Gambardella ML Luigiano C G LAT Scarlata GGM Luzza F Abenavoli L. Portal hypertension-associated gastric pathology: role of endoscopic banding ligation. Minerva Gastroenterol. (2025) 71:103–15. doi: 10.23736/S2724-5985.25.03759-3 40048148

[27] Yu LK Hsu CW Tseng JH Liu NJ Sheen IS. Splenic infarction complicated by splenic artery occlusion after N-butyl-2-cyanoacrylate injection for gastric varices: case report. Gastrointest Endosc. (2005) 61:343–5. doi: 10.1016/S0016-5107(04)02583-0 15729263

[28] Hwang SS Kim HH Park SH Kim SE Jung JI Ahn BY . N-butyl-2-cyanoacrylate pulmonary embolism after endoscopic injection sclerotherapy for gastric variceal bleeding. J Comput Assist Tomogr. (2001) 25:16–22. doi: 10.1097/00004728-200101000-00003 11176287

[29] Huang Z Zhang W Lv F Ma L Xiao Y Gao S . Efficacy and safety of EUS-guided coil embolization combined with endoscopic cyanoacrylate injection versus balloon-occluded retrograde transvenous obliteration for gastric varices with high-risk ectopic embolism: a multicenter and retrospective cohort study. Endosc Ultrasound. (2023) 12:74–83. doi: 10.4103/EUS-D-21-00260 36510863 PMC10134943

[30] Hu Y Zhou M Liu D Gong J. Risk factors for rebleeding after endoscopic injection of cyanoacrylate glue for gastric varices: a systematic review and meta-analysis. Dig Dis Sci. (2024) 69:2890–903. doi: 10.1007/s10620-024-08482-x 38864930

[31] Hakoshima M Kitakaze K Adachi H Katsuyama H Yanai H. Clinical, hematological, biochemical and radiological characteristics for patients with splenic infarction: case series with literature review. J Clin Med Res. (2023) 15:38–50. doi: 10.14740/jocmr4836 PMC9881492 36755765

[32] Boukobza M Rebibo L Ilic-Habensus E Iung B Duval X Laissy JP. Splenic abscess and infective endocarditis. Infection. (2025) 53:71–82. doi: 10.1007/s15010-024-02322-w 38916693

[33] Chikamori F Kuniyoshi N Shibuya S Takase Y. Urgent transjugular retrograde obliteration for prophylaxis of rebleeding from gastric varices in patients with a spontaneous portosplenorenal shunt. Dig Surg. (2000) 17:23–8. doi: 10.1159/000018796 10720828

